# Truncation or Deglycosylation of the Neuraminidase Stalk Enhances the Pathogenicity of the H5N1 Subtype Avian Influenza Virus in Mallard Ducks

**DOI:** 10.3389/fmicb.2020.583588

**Published:** 2020-10-22

**Authors:** Sujuan Chen, Keji Quan, Dandan Wang, Yinping Du, Tao Qin, Daxin Peng, Xiufan Liu

**Affiliations:** ^1^College of Veterinary Medicine, Yangzhou University, Yangzhou, China; ^2^Jiangsu Co-Innovation Center for the Prevention and Control of Important Animal Infectious Disease and Zoonoses, Yangzhou, China; ^3^Joint International Research Laboratory of Agriculture and Agri-Product Safety, The Ministry of Education of China, Yangzhou University, Yangzhou, China; ^4^Jiangsu Research Centre of Engineering and Technology for Prevention and Control of Poultry Disease, Yangzhou, China

**Keywords:** H5N1 subtype AIV, neuraminidase, glycosylation, pathogenicity, mallard duck

## Abstract

H5N1 subtype avian influenza virus (AIV) with a deletion of 20 amino acids at residues 49–68 in the stalk region of neuraminidase (NA) became a major epidemic virus. To determine the effect of truncation or deglycosylation of the NA stalk on virulence, we used site-directed mutagenesis to insert 20 amino acids in the short-stalk virus A/mallard/Huadong/S/2005 (SY) to recover the long-stalk virus (rSNA+). A series of short-stalk or deglycosylated-stalk viruses were also constructed basing on the long-stalk virus, and then the characteristics and pathogenicity of the resulting viruses were evaluated. The results showed that most of the short-stalk or deglycosylated-stalk viruses had smaller plaques, and increased thermal and low-pH stability, and a decreased neuraminidase activity when compared with the virus rSNA+. In a mallard ducks challenge study, most of the short-stalk or deglycosylated-stalk viruses showed increased pathological lesions and virus titers in the organ tissues and increased virus shedding in the oropharynx and cloaca when compared with the rSNA+ virus, while most of the short-stalk viruses, especially rSNA-20, showed higher pathogenicity than the deglycosylated-stalk virus. In addition, the short-stalk viruses showed a significantly upregulated expression of the immune-related factors in the lungs of the infected mallard ducks, including IFN-α, Mx1, and IL-8. The results suggested that NA stalk truncation or deglycosylation increases the pathogenicity of H5N1 subtype AIV in mallard ducks, which will provide a pre-warning for prevention and control of H5N1 subtype avian influenza in the waterfowl.

## Introduction

Avian influenza virus (AIV) has a wide range of hosts and can be isolated from poultry, waterfowl, and some mammals, in which it displays different morbidities and mortalities (Wheatley and Kent, 2015). The highly pathogenic avian influenza virus (HPAIV) of the H5N1 subtype leads to high morbidity and mortality in poultry, which is harmful to the poultry industry and thus results in huge economic losses. In addition, the H5N1 subtype AIV can infect humans and pose a huge threat to public health. Since HPAIV H5N1 infected humans and caused six deaths in Hong Kong in 1997, there have been many reports of H5N1 outbreaks in different regions, including Asia, Europe, and Africa (Obenauer et al., 2006; Jiang et al., 2017; Rashid et al., 2017). Data in as of May 08, 2020, more than 861 confirmed human H5N1 subtype virus infections have been reported, which have caused 455 deaths with a fatality rate of 52.7% (WHO, 2020).

AIV is extremely prone to antigen shift and drift, increasing the possibility of the virus adapting to humans (He et al., 2020). Alignment analysis of H5N1 subtype AIV isolated in recent years revealed that the number of viruses with a deletion of 20 amino acids at residues 49–68 of the neuraminidase (NA) stalk showed a continuous increase and became epidemic viruses (Zhou H. et al., 2009). Some researchers had speculated that the truncation of 20 amino acids is a molecular marker for the evolution of H5N1 subtype AIV from aquatic birds to terrestrial birds (Songserm et al., 2006; Zhou J. et al., 2009). NA is an important type II glycoprotein on the surface of AIV, which possesses exoglycosidase activity and helps to release progeny viruses by destroying sialic acid residues on the surface of the host cells (Medina and Garcia-Sastre, 2011). The glycosylation of NA plays an important role in protecting the stalk structure and strengthening immune escape (Paul et al., 2018). Although the mutation rate of NA glycosylation did not as fast as hemagglutinin (HA), this glycoprotein is still in a constantly changing dynamic process. For instance, there were four glycosylation sites (50NQS, 58NNT, 63NQT, and 68NIS) among the 20 amino acids deleted from the NA stalk, and these glycosylation sites disappeared with the truncation of the NA stalk (Chen et al., 2012).

The percentage of viruses with a short NA stalk dramatically increased from 1996 to 2012 among all avian isolates, including those from wild birds (Matsuoka et al., 2009). This has been reported previously in the NAs of avian influenza H2N2, H5N1, H6N1, H7N1, H7N9, H7N3, and H9N2 subtype viruses (Matrosovich et al., 1999; Banks et al., 2001; Campitelli et al., 2004; Matsuoka et al., 2009; Sorrell et al., 2010; Gao et al., 2013; Sun et al., 2013). Waterfowl are natural reservoirs of AIV, and 16 HA subtypes and 9 NA subtypes of AIV have been suggested to exist in wild waterfowl, most of which can be isolated from ducks (Shao et al., 2017). Therefore, ducks are vital in the epidemiology of AIV, playing an important role in the emergence and transmission of HPAIV H5N1 from waterfowl to terrestrial birds and humans (Yoon et al., 2014). H5N1 AIV was first isolated from diseased geese in Guangdong Province in 1996, which has spread to duck flocks in many areas of China (Chen et al., 2004; Bui et al., 2018). Although H5N1 AIV is highly virulent to most waterfowl, most free-range ducks show mild or no symptoms after H5N1 infection. However, after a long period of evolution, AIV has shifted the balance with its host and gained the ability to kill ducks, which are natural hosts for AIV (Saito et al., 2018). It has been reported that the deletion in the NA stalk effectively enhanced the cell adaptability of H5N1 AIV, and the amount of virus released from MDCK cells infected with the short-stalk virus was 10–100 times higher than that produced by the intact virus (Wang et al., 2006). However, the role of the deletions or deglycosylation in the NA proteins on the pathogenicity of the H5N1 subtype AIVs in mallard ducks remains unknown. Given the prevalence of different subtypes of AIV in China, it is necessary to further study the molecular changes in the stalk region of NA. Hence, we investigated the effects of NA stalk truncation or deglycosylation on viral pathogenicity in mallard ducks.

In this study, we constructed a series of NA stalk-truncated or deglycosylated recombinant viruses by reverse genetics and determined their biological characteristics, including replication kinetics, stability under different conditions, neuraminidase activity, and pathogenicity in mallard ducks.

## Materials and Methods

### Ethics Statement

H5, H7, and H9 AIV-free mallard ducks, used as an animal model in the present study, were purchased from a standardized farm. All experiments involving live H5 viruses were approved by the Institutional Biosafety Committee of Yangzhou University and were performed in animal biosafety level 3 (ABSL-3) facilities according to the institutional biosafety manual (CNAS BL0015). The protocols for all animal studies were approved by Jiangsu Province Administrative Committee for Laboratory Animals (approval number: SYXK-SU-2017-0007) and complied with the guidelines of Jiangsu Province Laboratory Animal Welfare and the Ethics of Jiangsu Province Administrative Committee of Laboratory Animals.

### Viruses and Cells

HPAIV H5N1 A/mallard/Huadong/S/2005 (SY, EU195394.1), with a 20-amino acid deletion in the NA stalk, was isolated from mallard ducks in eastern China (Tang et al., 2008). Human embryonic kidney (293T) and MDCK cells were grown in Dulbecco’s modified Eagle’s medium (DMEM, HyClone, United States) supplemented with 10% fetal bovine serum (FBS, Gibco, United States) plus antibiotics. Chicken embryo fibroblasts (CEF) and duck embryo fibroblasts (DEF) were grown in M199 medium (HyClone, United States) with 4% FBS. The cells were incubated at 37°C with 5% CO_2_.

**FIGURE 1:**
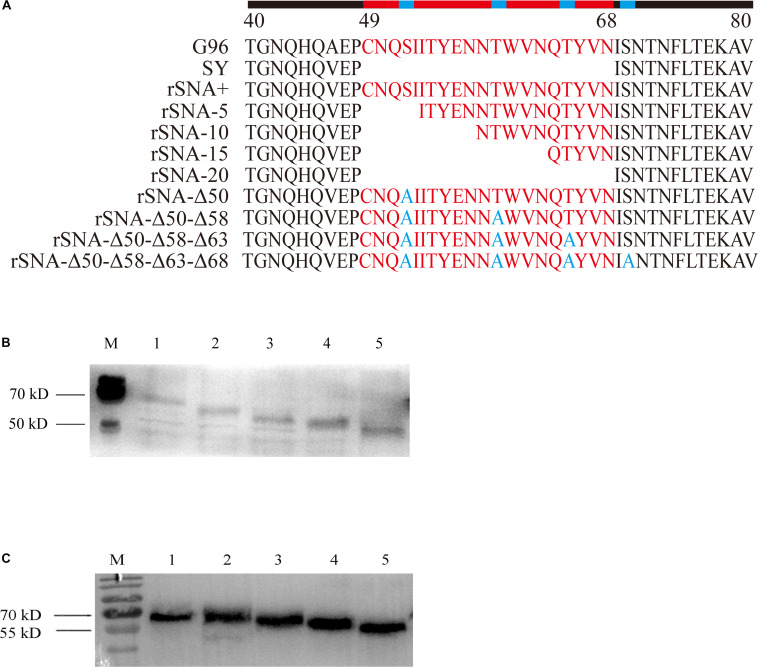
SY NA stalks mutant viruses and their western blot analysis. **(A)** SY and their NA stalk mutant viruses were generated by reverse genetics based on the NA stalk truncation or deglycosylation strategy. **(B)** Four recombinant viruses with different glycosylation patterns in the stalk of the NA and rSNA+. 1–5: rSNA+, rSNA-Δ50, rSNA-Δ50–Δ58, rSNA-Δ50–Δ58–Δ63, and rSNA-Δ50–Δ58–Δ63–Δ68. **(C)** Four recombinant viruses with the shortened stalk NA and rSNA+. 1–5: rSNA+, rSNA-5, rSNA-10, rSNA-15, and rSNA-20.

For virus mutagenesis, the stalk-deleted and glycosylation site-deleted viruses, including rSNA-5, rSNA-10, rSNA-15, rSNA-20, rSNA-Δ50, rSNA-Δ50-Δ58, rSNA-Δ50-Δ58-Δ63, rSNA-Δ50-Δ58-Δ63-Δ68, and complete-stalk virus rSNA+, were constructed with the insertion of the sequence from a H5N1 subtype AIV, termed A/Goose/Guangdong/1/96 (G96, NC_007361.1), at residues 48–69 of the NA stalk into the parental virus SY by reverse genetic manipulations (Figure 1A). In brief, mutant primers were matched (Table 1) to Hoffmann and Webster (2001) NA primers and the amplified fragments were cloned into the pHW2000 plasmid to obtain pHW-NA-5, pHW-NA-10, and pHW-NA-15. The pHW-NA+ and pHW-NA-20 were previously constructed (Tang Y. et al., 2009; Li et al., 2014). Then, with pHW-NA+ as a template for point mutation, primers (Table 1) were designed according to the manual of the Mut Express^®^ MultiS Fast Mutagenesis Kit V2 (Vazyme, China). To construct the plasmids (pHW-NA-Δ50, pHW-NA-Δ50-Δ58, pHW-NA-Δ50-Δ58-Δ63, and pHW-NA-Δ50-Δ58-Δ63-Δ68) with different glycosylation site deletions, the S/T was mutated to A in the N-X-S/T glycosylation sequences. The recombinant plasmids were co-transfected into 293T plus MDCK cells (3:1) with the SY eight plasmids system (Tang et al., 2008) according to the transfection method by Hoffmann et al. (2002) and Shi et al. (2007). The allantoic fluid was assessed using a hemagglutination assay with 1% chicken red blood cells (CRBCs).

**TABLE 1:** Primers for construction of mutant plasmids of NA genes.

Target gene	Primer name	Primer sequences (5′ → 3′)
	Ba-NA-1	TATT***GGTCTC***AGGGAGCAAAAGCAGGAGT
	Ba-NA-2	ATAT***GGTCTC***GTATTAGTAGAAACAAGGAGTT TTTT
NA+	NA+-R	TATGTCTGATTTACCCAGGTGTTGTTTTCATAA GTAATAATGCTTTGATTGCATGGTTCAACTTGG TGTTGATTCCCTGTCTGAATT
	NA+-F	ACTTATGAAAACAACACCTGGGTAAATCAGAC ATATGTCAACATCAGCAATACTAATTTTCTTAC TGAGAAAGCTGTGGCTT
NA-5 aa	NA-5-R	CATAAGTAATTGGTTCAACTTGGTGTT
	NA-5-F	CACCAAGTTGAACCAATTACTTATG
NA-10 aa	NA-10-R	TTACCCAGGTGTTTGGTTCAACTTG
	NA-10-F	ACCAAGTTGAACCAAACACCTG
NA-15 aa	NA-15-R	TATGTCTGTGGTTCAACTTGGTGTT
	NA-15-F	CACCAAGTTGAACCACAGACATATGTCAAC
NA-Δ50	50-F	ATGCAATCAAGCCATTATTACTTATGAAAAC
	50-R	GTAATAATGGCTTGATTGCATGGTTCAACTT
NA-Δ58	58-F	TGAAAACAACGCCTGGGTAAATCAGGCATATG
	58-R	GATTTACCCAGGCGTTGTTTTCATAAGTAATAAT
NA-Δ63	63-F	CACCTGGGTAAATCAGGCATATGTCAACAT
	63-R	TATTGCTGATGTTGACATATGCCTGATTTAC
NA-Δ68	68-F	ATGTCAACATCGCCAATACTAATTTTCTTACTGAG
	68-R	TTAGTATTGGCGATGTTGACATATGTCTGATTTAC

***GGTCTC** is the recognition site of BsaI. The change of nucleotides is underlined.*

### Western Blot Analysis

The CEF cells were infected with the recombinant viruses at a multiplicity of infection (MOI) of 1 in M199 medium. The infected cells were washed three times with PBS to remove unbound virus particles, after which the fresh M199 was added. At 24 h post-infection (p.i.), the cells were lysed with 200 μl whole-cell lysis buffer (Beyotime, China) on ice for 30 min. The lysates were collected with a scraper, and the whole-cell extract was harvested by centrifugation at 13,000 rpm, 4°C for 10 min. The proteins were loaded into lanes on SDS-PAGE gels and transferred to a nitrocellulose membrane. The membrane was blocked in 5% skimmed milk and then incubated with a polyclonal antibody against NA (anti-NA of H5N1 subtype AIV), followed by incubation with horseradish peroxidase-conjugated goat anti-mouse IgG antibodies (Sigma, Germany). After washing three times, the protein bands were detected with Electrochemiluminescence (ECL) (Thermo, United States).

### Virus Growth

The CEF and DEF cells were infected in triplicate with the recombinant viruses at an MOI of 0.01 in M199, and the medium was discarded at 1 h.p.i. The virus titers of the supernatants, which were collected at different time points, were quantified as the 50% tissue culture infectious doses (TCID_50_) per 1 ml of the CEF cell culture by using the method described by Reed and Muench (1937).

For the plaque assays, MDCK cells were seeded into six-well plates and incubated. The monolayer cells were infected with 10-fold dilutions of the recombinant viruses in a total volume of 500 μl of serum-free DMEM. After incubation for 1 h, the unbound viruses were removed, and the cells were washed with PBS and then overlaid with DMEM containing 0.8% agar (Sigma, Germany) and 2% FBS. The infected cells were incubated for 72 h, and the plaques were visualized by staining with 0.1% crystal violet in a 10% formaldehyde solution. For each virus, 10 well-spaced plaques were measured using the GNU image manipulation program (version 2.8^1^).

### Thermostability and Low-pH Stability

The viruses were diluted to 10^8^ TCID_50_/ml and aliquoted into vials. The viruses were incubated at 37°C or 42°C for 1, 3, and 5 days, the samples were temporarily frozen at −70°C before all the samples were collected, and the TCID_50_ values were determined on the CEF cells. The recombinant viruses were mixed with 100 mM acetate buffer (pH 4.0 and pH 5.0), 100 mM phosphate buffer (pH 6.0) or PBS (pH 7.0) and incubated at 37°C for 10 min, after which their hemagglutination titers were determined.

### Release of Virus From Erythrocytes and Neuraminidase Activity

To determine the rate of virus release from the CRBCs was assessed as described previously (Castrucci and Kawaoka, 1993). Briefly, 50 μl of twofold serial dilutions of the viral stocks in PBS was incubated with 50 μl 1% CRBC suspension in microtiter plates at 4°C for 1 h, after which the plates were stored at 37°C. The hemagglutination assay titer, which reflects the NA-mediated virus release from the CRBCs, was monitored for 12 h.

The NA activity was determined by a fluorescence-based assay using a Neuraminidase Assay Kit (Beyotime, China) according to the manufacturer’s instructions. In brief, 10 μl of allantoic fluid was added to 70 μl of detection buffer, followed by adding 10 μl of NA fluorogenic substrate and 10 μl of ultrapure water. After incubation at 37°C for 1 h, the cleavage of the NA fluorogenic substrate produced fluorescence (excitation wavelength of 322 nm and emission wavelength of 450 nm), which was measured by using a fluorescence spectrophotometer (BioTek, United States). The NA activity was indicated as the fluorescence intensity of the samples.

### Pathogenicity in Mallard Ducks

To determine the pathogenicity of the recombinant viruses in mallard ducks, four-week-old mallard ducks (*n* = 90) were randomly divided into 10 groups and inoculated intranasally with 10^6.0^ EID_50_ of the respective recombinant viruses (rSNA-Δ50, rSNA-Δ50–Δ58, rSNA-Δ50–Δ58–Δ63, rSNA-Δ50–Δ58–Δ63–Δ68, rSNA+, rSNA-5, rSNA-10, rSNA-15, and rSNA-20). The controls were mock-infected with PBS. Clinical symptoms were assessed daily for 7 d.p.i. Three ducks in each group were sacrificed at 3, 5, and 7 d.p.i. The general pathological changes in the tissues were observed. The viral load of the heart, spleen, lung, kidney, and brain was measured, the tissues were fully ground according to the standard of 1 g tissue sample/3 ml PBS. The samples were freeze-thawing three times to make the virus fully released, then the supernatant was diluted to 10^–6^ by a continuous 10-fold gradient and inoculated with chicken embryos. The allantoic fluid was taken for hemagglutination assay, and the detection line was 10^1^.^48^ EID_50_/ml. At the same time, cloaca and oropharyngeal swabs of all the surviving ducks were collected to determine the virus shedding. Three ducks were euthanized in each group on 3, 5, and 7 d.p.i.

### Histopathologic Analysis

Four-week-old mallard ducks (*n* = 3/group) were intranasally inoculated with the allantoic fluid of one of the nine recombinant viruses diluted with PBS to 10^6.0^ EID_50_, while the control group was inoculated with PBS. On 5 d.p.i., three ducks from each group were euthanized. The heart, spleen, lung, kidney, and brain tissues were collected and fixed in 10% neutral buffered formalin overnight, after which they were processed routinely, embedded in paraffin, sectioned into 4 μm slices, stained with hematoxylin and eosin, and observed microscopically for histopathologic alterations (McAuley et al., 2007). In the histopathology of the hearts, brains, lungs, spleens, and kidneys of the mallard ducks, the number of “+” represents the degree of the lesion, the details as follows. + includes mild bleeding, congestion, and a small amount of inflammatory cell infiltration. ++ includes moderate necrosis, moderate inflammatory cell infiltration, and a significant increase in the number of red blood cells. +++ includes vascular cuff phenomenon, and severe bleeding. ++++ includes severe necrosis focus, massive inflammatory cell infiltration, and severe bleeding or congestion (Table 2 and Supplementary Figure 1).

**TABLE 2:** Morphometric analysis of histopathological lesion score.

Group	The degree of the lesion				
	Heart	Brain	Lung	Spleen	Kidney
rSNA-Δ50	++++	++	++	+	+
rSNA-Δ50–Δ58	++	++	+	+	+
rSNA-Δ50–Δ58–Δ63	++	+	++	++	+
rSNA-Δ50–Δ58–Δ63–Δ68	+	+	+++	+	+
rSNA+	+	–	++	+++	+
rSNA-5	++++	++	+++	+++	+
rSNA-10	++	++	+++	++++	+
rSNA-15	++++	++++	++++	–	+
rSNA-20	++++	++++	++++	+++	+

*The number of “+” represents the degree of the lesion, the more “+” the more serious. “–” no obvious histopathologic changes were observed.*

### Quantitative Real-Time PCR (qRT-PCR)

Quantitative real-time PCR (qRT-PCR) was used to analyze the innate immune response-related representative cytokine genes. Total RNA was isolated from tissue using the HP Total RNA Kit (OMEGA, United States). The RNA of each sample was reverse transcribed into cDNA using Prime Script^TM^ RT reagent Kit with gDNA Eraser (TAKARA, Japan). The primers for the IFN-α, Mx1, IL-6, IL-8, IL-10, and IL-1β genes were designed according to the published sequences or previously reported primers (Hu et al., 2013). All of the primers are listed in Table 3. The expression level of each gene relative to that of GAPDH was calculated using the 2^–ΔΔCT^ method (Livak and Schmittgen, 2001).

**TABLE 3:** Primers of qRT-PCR for detection of cytokine mRNA level in the lungs of mallard ducks.

Target gene	Forward primer	Reverse primer
GAPDH	ATGTTCGTGATGGGTGTGAA	CTGTCTTCGTGTGTGGCTGT
M×1	TCACACGAAGGCCTATTTTACTGG	GTCGCCGAAGTCATGAAGGA
IL-10	GGGGAGAGGAAACTGAGAGATG	TCACTGGAGGGTAAAATGCAGA
IL-1β	GAGATTTTCGAACCCGTCACC	AGGACTGGGAGCGGGTGTA
IL-8	AGGACAACAGAGAGGTGTGCTTG	GCCTTTACGATCCGCTGTACC
IL-6	CTGGCTTCGACGAGGAGAAA	CGTCGTTGCCAGATGCTTTG
IFN-α	TTGCTCCTTCCCGGACA	GCTGAGGGTGTCGAAGAGGT

### Statistical Analysis

SPSS 19.0 (IBM Inc., United States) was used for statistical analysis of the data; the difference between the two groups was analyzed using the Student’s *t*-test, and the difference among three groups or more was statistically compared by ANOVA. Pearson’s chi-square test or Fisher’s exact test was used to compare the difference in the swab positivity rate. The software GraphPad Prism version 6 (GraphPad Software, San Diego, CA, United States) was used to make the graphs. Statistical significance was denoted by the symbols ^∗^ (*P* < 0.05) and ^∗∗^ (*P* < 0.01) for comparison with the results for virus rSNA+. The data are shown as the mean fold change ± SD of the results.

## Results

### Generation of Recombinant Viruses With Different Patterns in the NA Stalk Region

There was no spontaneous mutation that occurred during the rescue and amplification procedure of all recombinant viruses (Figure 1A). Western blot results showed that compared with rSNA+, the molecular weight of NA from the recombinant virus was reduced with the degrees of stalk truncation or deglycosylation. These results proved that the recombinant viruses were successfully constructed (Figures 1B,C).

### Replication Kinetics of Recombinant Viruses With Different Patterns in the NA Stalk Region

**FIGURE 2:**
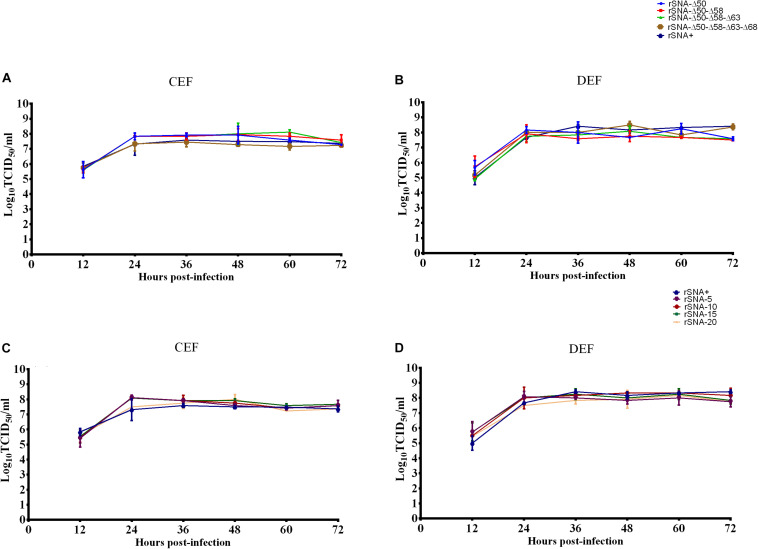
Growth curves of recombinant viruses in CEF **(A,C)** and DEF **(B,D)** cells. The mean and standard errors are shown from three independent experiments.

It was determined that the nine recombinant AIVs were replicated efficiently on the CEF and DEF cells. There was no significant difference in the growth rate of each recombinant virus on the CEF and DEF cells (Figure 2).

**FIGURE 3:**
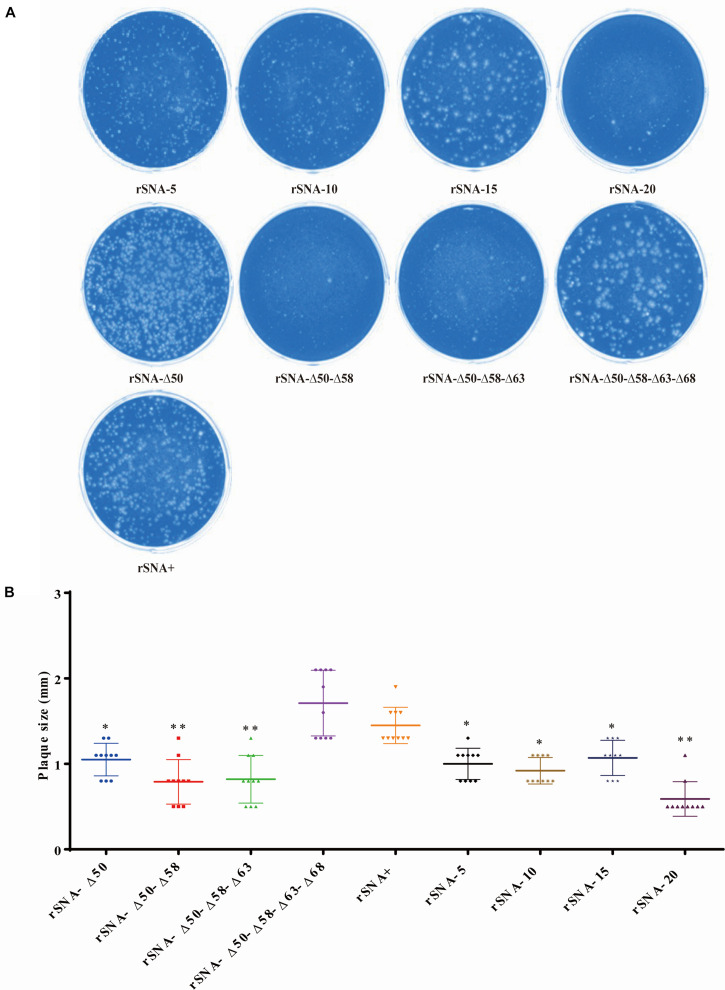
Plaque assay in MDCK cells of recombinant viruses **(A)**. The diameter of 10 independent plaques was measured for each virus sample **(B)**. *P < 0.05, **P < 0.01, compared to the value of the rSNA+.

The plaque diameter of complete-stalk virus rSNA+ was significantly larger than that of the others (*P* < 0.05), except for a glycosylation-deleted virus rSNA-Δ50–Δ58–Δ63–Δ68. In addition, plaque diameters of deglycosylation viruses, including rSNA-Δ50–Δ58 and rSNA-Δ50–Δ58–Δ63, or stalk-deleted AIV rSNA-20, were significantly less than that of rSNA+ (*p* < 0.01) (Figures 3A,B).

### Thermostability and Low-pH Stability of Recombinant Viruses With Different Patterns in the NA Stalk Region

**FIGURE 4:**
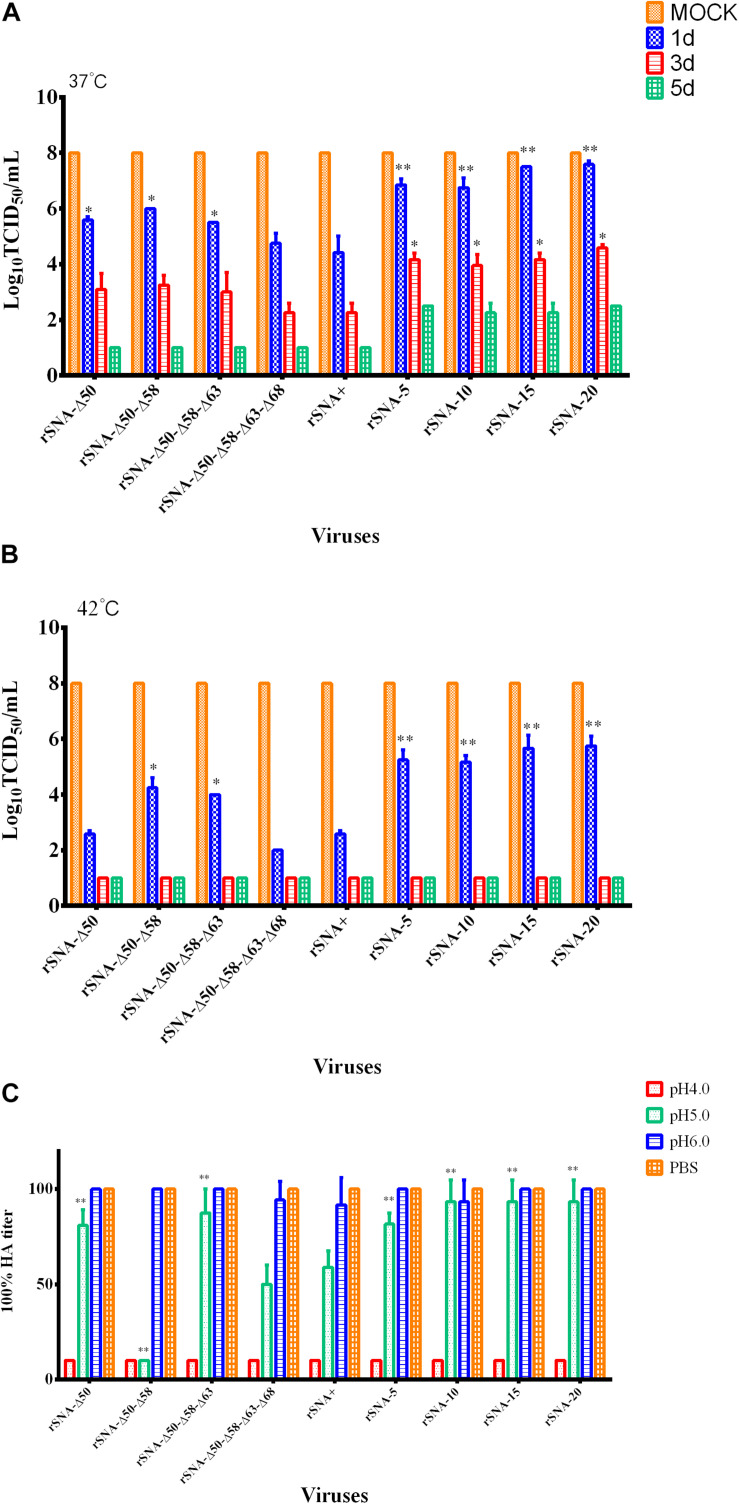
Thermal stability of the recombinant viruses at 37°C **(A)**, 42°C **(B)**, and low-pH stability of them **(C)**. *P < 0.05, **P < 0.01, compared to the value of the rSNA+.

When incubation at 37°C for 1 day, the rSNA-Δ50, rSNA-Δ50–Δ58, and rSNA-Δ50–Δ58–Δ63 exhibited enhanced stability compared with rSNA+ (*P* < 0.05). After incubation at 37°C for 1 and 3 days, the infectivity of the complete-stalk viruses was lower than those of the stalk-deleted AIVs. When incubated at 37°C for 5 days, all complete-stalk viruses were completely inactivated, while the stalk-deleted viruses remained infectious (Figure 4A). At 42°C for 1 day, the infectivity of rSNA-Δ50–Δ58 and rSNA-Δ50–Δ58–Δ63 were higher than that of rSNA+ (*P* < 0.05), and the abilities of all stalk-deleted viruses to infect the CEF cells were higher than that of rSNA+ (*P* < 0.01). At 42°C for 3 days, all the recombinant viruses were completely inactivated (Figure 4B). Therefore, the thermostability of the recombinant viruses with the shortened stalk NA was better than that of rSNA+ and the viruses with different glycosylation patterns in the stalk region of the NA. The thermostability of glycosylation-deficient strains was not significantly different in comparison to that of the intact strain, however, there was a difference in low-pH stability. rSNA-Δ50 and rSNA-Δ50–Δ58–Δ63 significantly improved the stability, while rSNA-Δ50–Δ58 showed a very significant decrease in the stability of low-pH. Additionally, the simultaneous deletion of four glycosylation sites had no significant effect on the stability of low-pH.

The low-pH stability assay results showed that all viruses were inactivated at pH 4.0 (Figure 4C). The HA titers of all recombinant AIVs were decreased at pH 5.0, and rSNA-Δ50–Δ58 had a significant decrease compared with rSNA+ (*P* < 0.01). The rSNA-Δ50, rSNA-Δ50–Δ58–Δ63, and all viruses with the shortened-stalk NA exhibited enhanced stability compared with rSNA+ at pH 5.0 (*P* < 0.01). The HA titers of rSNA+ and rSNA-Δ50–Δ58–Δ63–Δ68 showed a decrease compared with that of rSNA-20 at pH 5.0. The results indicated that the deletion of the NA stalk region improved the stability at low-pH.

### Virus Release Ability and Neuraminidase Activity of Recombinant Viruses With Different Patterns in the NA Stalk

**FIGURE 5:**
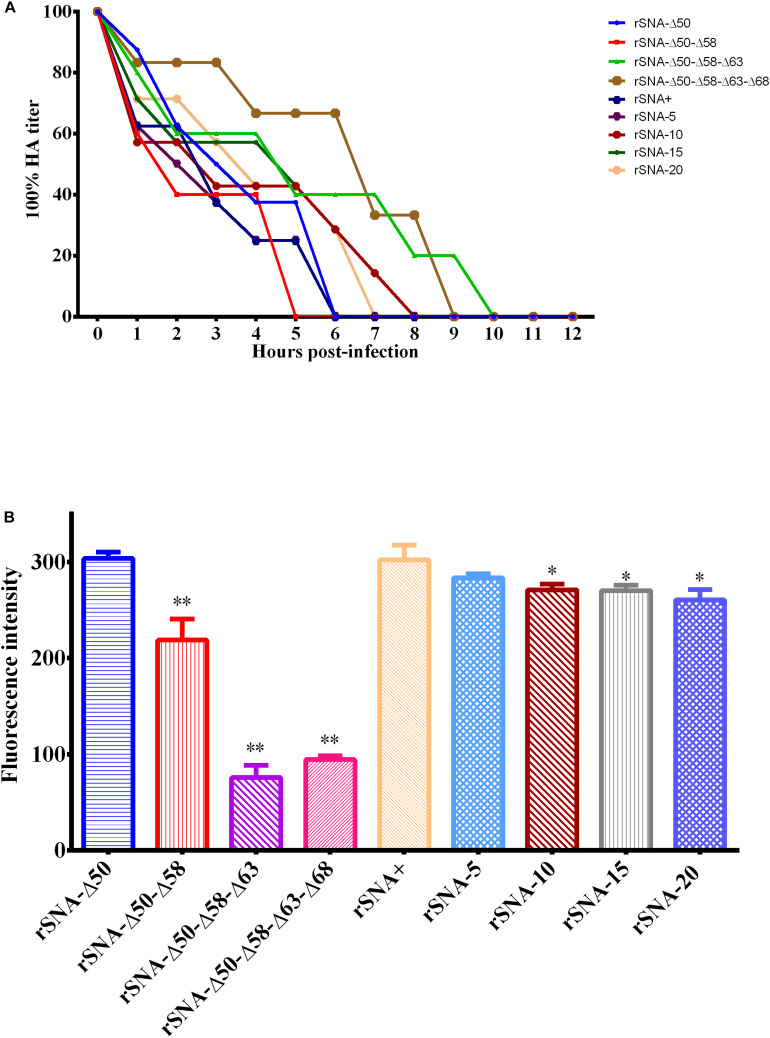
Curves of the recombinant viruses released from the red blood cell surface **(A)** and neuraminidase activity assays of them **(B)**. *P < 0.05, **P < 0.01, compared to the value of the rSNA+.

The results of the viral release from the CRBCs demonstrated that rSNA-Δ50–Δ58, released completely within 5 h, which had the fastest release rate, and all recombinant viruses released from the surface of the CRBCs within 10 h (Figure 5A). The results of the neuraminidase activity assay showed that the activity of rSNA+ was the highest, the activity of the mutant strains was decreased compared with the NA-intact virus, and the decrease in the deglycosylated viruses was more significant (*P* < 0.01), except for rSNA-Δ50 (Figure 5B).

### Pathogenicity of Recombinant Viruses With Different Patterns in the NA Stalk in Mallard Ducks

When intranasal inoculation with a dose of 10^6.0^ EID_50_, all viruses were able to be detected in the heart, spleen, lung, kidney, and brain of mallard ducks (Table 4). At 3 d.p.i., the mean viral titers in the heart, lung, kidney, and spleen of the two groups infected with viruses with a truncated stalk of the NA (rSNA-15 and rSNA-20) and all of the recombinant viruses with the different deglycosylation sites were higher than that of the rSNA+ group. At 5 d.p.i., the mean viral titers in all organs of animals infected with the four shortened-stalk NA recombinant viruses were higher than those of animals infected with the rSNA+ and the four recombinant AIVs with the different glycosylation patterns in the stalk of the NA, with one exception, the titer of rSNA-10 was not higher than rSNA-Δ50 in heart. At the same time, the mallard ducks infected with the viruses with the truncated NA stalk showed clinical symptoms, and some of the mallard ducks infected with rSNA-5, rSNA-15, and rSNA-20 showed neurological symptoms, and some occurred death in the rSNA-5 and rSNA-15 groups, which implied that the truncated NA stalk might enhance the pathogenicity of the H5N1 subtype AIV to the mallard duck nervous system.

**TABLE 4:** Replication of recombinant viruses in the experimentally infected mallard ducks.

Group	Heart			Spleen			Lung			Kidney			Brain		
	3 d.p.i.	5 d.p.i.	7 d.p.i.	3 d.p.i.^b^	5 d.p.i.	7 d.p.i.	3 d.p.i.	5 d.p.i.	7 d.p.i.	3 d.p.i.	5 d.p.i.	7 d.p.i.	3 d.p.i.	5 d.p.i.	7 d.p.i.
rSNA-Δ50	1/3 (4.0 ± 0)^a^	2/3 (3.9 ± 1.0)	2/3 (2.7 ± 1.0)	1/3 (4.2 ± 0)	2/3 (3.1 ± 0.2)	2/3 (3.7 ± 0.7)	1/3 (4.7 ± 0)	2/3 (5.2 ± 0.7)	3/3 (4.0 ± 1.0)	1/3 (5.0 ± 0)	2/3 (4.6 ± 1.2)	3/3 (3.4 ± 1.1)	1/3 (4.0 ± 0)	2/3 (4.1 ± 1.2)	2/3 (2.7 ± 05)
rSNA-Δ50–Δ58	2/3 (3.7 ± 0.4)	2/3 (3.0 ± 0.9)	0/3	2/3 (4.5 ± 0.4)	3/3 (4.1 ± 0.9)	0/3	2/3 (5.0 ± 0.4)	3/3 (4.6 ± 1.2)	0/3	2/3 (5.5 ± 0.1)	3/3 (4.3 ± 0.9)	0/3	1/3 (2.2 ± 0)	3/3 (3.9 ± 0.9)	0/3
rSNA-Δ50– Δ58–Δ63	3/3 (3.7 ± 0.3)	2/3 (2.5 ± 0.4)	0/3	3/3 (5.6 ± 0.4)	2/3 (3.0 ± 1.1)	0/3	3/3 (5.3 ± 0.6)	3/3 (4.1 ± 0.5)	0/3	3/3 (5.1 ± 0.3)	2/3 (3.0 ± 1.1)	1/3 (3 ± 0)	3/3 (4.1 ± 0.8)	2/3 (2.5 ± 0.4)	0/3
rSNA-Δ50– Δ58–Δ63– Δ68	3/3 (4.1 ± 0.7)	3/3 (2.7 ± 0.7)	0/3	3/3 (5.3 ± 0.6)	2/3 (2.9 ± 0.9)	0/3	3/3 (5.3 ± 0.6)	2/3 (3.9 ± 1.1)	1/3 (3 ± 0)	3/3 (5.6 ± 0.5)	2/3 (3.5 ± 0.4)	0/3	3/3 (4.3 ± 1.2)	2/3 (3.5 ± 0.3)	1/3 (3 ± 0)
rSNA+	1/3 (3.2 ± 0)	3/3 (3.4 ± 0.8)	0/3	1/3 (3.5 ± 0)	2/3 (3.9 ± 0.2)	0/3	2/3 (4.5 ± 0.5)	2/3 (4.9 ± 0.2)	0/3	1/3 (4.5 ± 0)	2/3 (4.0 ± 0.4)	0/3	1/3 (4.0 ± 0)	3/3 (2.9 ± 0.6)	0/3
rSNA-5	2/3 (4.2 ± 1.1)	3/3 (4.3 ± 0.6)	0/3	1/3 (3.2 ± 0)	3/3 (4.1 ± 1)	0/3	2/3 (4.7 ± 0)	3/3 (5.6 ± 0.5)	0/3	1/3 (5.0 ± 0)	3/3 (4.7 ± 0.7)	0/3	1/3 (4.5 ± 0)	3/3 (5.3 ± 0.4)	0/3
rSNA-10	3/3 (2.7 ± 0.7)	2/3 (3.0 ± 0)	1/3 (3 ± 0)	3/3 (3.4 ± 0.9)	2/3 (4.4 ± 0.9)	1/3 (2.7 ± 0)	3/3 (4.5 ± 0.4)	2/3 (5.5 ± 0.5)	1/3 (3 ± 0)	3/3 (3.9 ± 0.6)	2/3 (5.4 ± 0.9)	2/3 (2.4 ± 0.5)	3/3 (3.1 ± 0.9)	2/3 (5.5 ± 0.2)	1/3 (1.7 ± 0)
rSNA-15	2/3 (3.6 ± 05)	2/3 (4.9 ± 0.2)	0/3	3/3 (4.6 ± 0)	2/3 (4.4 ± 0.5)	0/3	3/3 (4.6 ± 1.2)	2/3 (6.4 ± 0.9)	2/3 (2.7 ± 0)	3/3 (4.7 ± 1.1)	2/3 (5.7 ± 0)	0/3	2/3 (4.2 ± 1.2)	3/3 (5.9 ± 0.2)	0/3
rSNA-20	3/3 (4.1 ± 0.1)	2/3 (5.4 ± 0.5)	0/3	3/3 (4.0 ± 0)	2/3 (4.2 ± 0.4)	0/3	3/3 (6.0 ± 0)	2/3 (6.3 ± 0.4)	1/3 (3 ± 0)	3/3 (4.5 ± 0.4)	2/3 (5.1 ± 0.2)	0/3	3/3 (4.6 ± 0.7)	2/3 (6.0 ± 0.2)	0/3

*^a^No. virus-positive mallard ducks/no. tested mallard ducks (log_10_EID_50_/mL ± SD).*

At 3, 5, and 7 d.p.i., the recombinant viruses can be detected in both the oropharynx and the cloaca, with the oropharynx as the main virus shedding route. Compared with the ducks infected with rSNA+, recombinant AIVs that had truncations or different glycosylation patterns in the stalk of the NA showed higher viral shedding. The viral shedding ratios of the recombinant viruses with the shortened stalk were higher than those of the deglycosylation viruses (Table 5). Therefore, deletion of the glycosylation sites or the NA stalk regions increased the viral shedding rate, but the effect from the stalk deletions was more significant.

**TABLE 5:** The viral positive ratio of oropharyngeal and cloacal swabs.

Group	Oropharyngeal swabs			Cloacal swabs		
	3 d.p.i.	5 d.p.i.	7 d.p.i.	3 d.p.i.	5 d.p.i.	7 d.p.i.
rSNA-Δ50	55.6%(5/9)^b^	66.7%(4/6)	0%(0/3)	22.2%(2/9)	16.7%(1/6)	0%(0/3)
rSNA-Δ50–Δ58	44.4%(4/9)	66.7%(4/6)	0%(0/3)	33.3%(3/9)	16.7%(1/6)	0%(0/3)
rSNA-Δ50–Δ58–Δ63	44.4%(4/9)	50%(3/6)	0%(0/3)	22.2%(2/9)	16.7%(1/6)	0%(0/3)
rSNA-Δ50–Δ58–Δ63–Δ68	88.9%(8/9)	83.3%(5/6)	33.3%(1/3)	44.4%(4/9)	66.7%(4/6)	33.3%(1/3)
rSNA+	44.4%(4/9)	33.3%(2/6)	0%(0/3)	11.1%(1/9)	16.7%(1/6)	0%(0/3)
rSNA-5	77.8%(7/9)	83.3%(5/6)	0%(0/3)	44.4%(4/9)	50%(3/6)	0%(0/3)
rSNA-10	66.7%(6/9)	66.7%(4/6)	0%(0/3)	33.3%(3/9)	16.7%(1/6)	0%(0/3)
rSNA-15	100%(9/9)^a^	83.3%(5/6)	0%(0/3)	66.7%(6/9)^a^	33.3%(2/6)	0%(0/3)
rSNA-20	88.9%(8/9)	66.7%(4/6)	0%(0/3)	44.4%(4/9)	33.3%(2/6)	0%(0/3)

*^a^P < 0.05 compared to the value of the rSNA+. ^b^No. positive swabs/no. tested swabs.*

In the histopathology of the hearts, brains, lungs, spleens, and kidneys of the mallard ducks, the number of “+” represents the degree of the lesion (Table 2 and Supplementary Figure 1). In the heart and brain tissue, the NA stalk-deleted and the deglycosylation AIVs caused more severe damage than the rSNA+, while in the lungs, the deglycosylation viruses induced milder symptoms than the four short-stalk viruses. In the spleens, the deglycosylation viruses induced milder symptoms than the truncation viruses except for rSNA-15. In all challenged groups, different degrees of myocardial fibrous necrosis were observed in the heart tissues of the ducks, with varying numbers of lymphocytes and macrophages infiltrating in and around the necrotic foci. Mallard ducks of all infection groups had different degrees of dilatation of the blood vessels in the brain tissues, filled with different numbers of red blood cells, the phenomenon of “vascular mantle,” formed by lymphocytes and macrophages, was observed around small vessels, and different degrees of lymphocyte infiltration can be seen under the meninges of the brain. In all infected groups, different degrees of hemorrhage, congestion, necrosis, edema, and infiltration of lymphocytes and macrophages were observed in the lungs. In all infected groups, the spleens exhibited varying degrees of hemorrhage or congestion, accompanied by a decrease in the number of lymphocytes and an unequal number of heterophilic granulocytes in the splenic sinus. In all infected groups, the kidneys showed different degrees of congestion, while there was no significant difference among the groups. Overall, the pathological damage caused by the truncated NA viruses in all tissues was more severe.

### Recombinant Virus-Induced Changes in Immune-Related Factors

**FIGURE 6:**
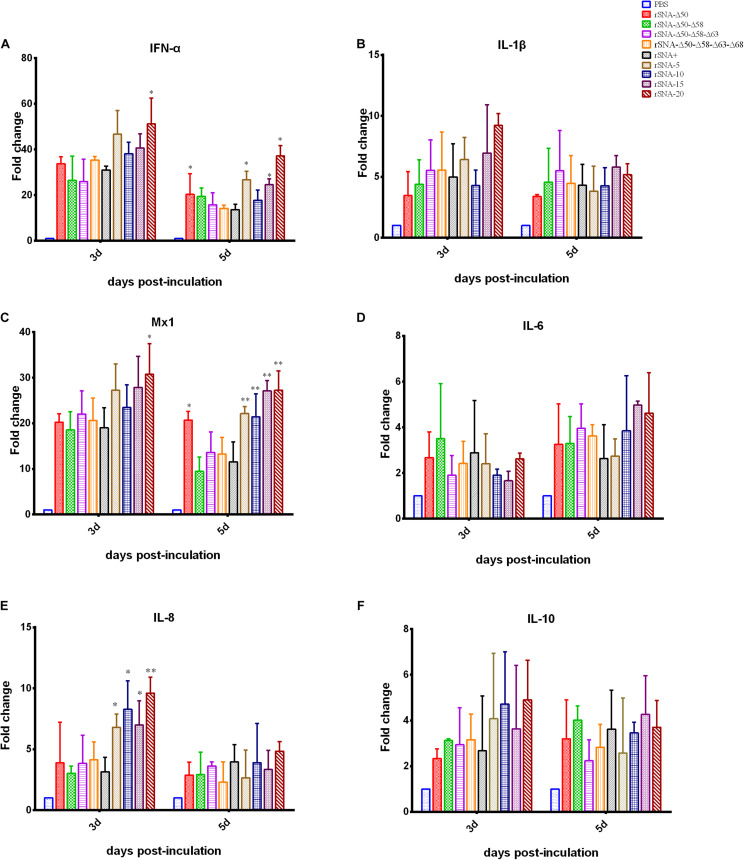
Cytokine response in the lung of the virus-infected mallard ducks. **(A)** IFN-α, **(B)** IL-1β, **(C)** Mx1, **(D)** IL-6, **(E)** IL-8, and **(F)** IL-10. *P < 0.05, **P < 0.01, compared to the value of the rSNA+.

All of the nine viruses can upregulate the expression of immune-related factors in the lungs of mallard ducks (Figure 6). At 3 d.p.i., rSNA-20 induced significantly higher levels of IFN-α and Mx1 expression than rSNA+ in the lungs (*P* < 0.05). Meanwhile, the four recombinant viruses with the shortened NA stalk induced significantly higher levels of IL-8 expression in the lungs than rSNA+ (*P* < 0.05). At 5 d.p.i., rSNA-Δ50, rSNA-5, rSNA-15, and rSNA-20 induced significantly higher levels of IFN-α expression than rSNA+ in the lungs (*P* < 0.05). rSNA-Δ50 and all viruses with the shortened NA stalk induced significantly higher levels of Mx1 expression than rSNA+ in the lungs (*P* < 0.01). Although, there were no significant differences that the levels of IL-1β, IL-6, and IL-10 induced by recombinant viruses (*P* > 0.05), it also showed the same upregulated trend with other immune factors. Therefore, the rSNA-20 can significantly upregulate the expression of IFN-α, Mx1, and IL-8.

## Discussion

Waterfowl have been regarded as the natural reservoir for AIVs, and most AIV subtypes exhibit a low pathogenicity in ducks (Venkatesh et al., 2018). However, it has been reported that the H5N1 subtype AIV has high pathogenicity in waterfowl. AIV recessively infects ducks without any symptoms, however, more strains have acquired the ability to kill their natural host ducks along with the viral evolution (Yoon et al., 2014). With the cross-transmission of AIVs occurring in birds and mammals, humans are at risk at any time of contracting the zoonotic pathogens (Lloren et al., 2017; Bergervoet et al., 2019; Song et al., 2019). In any case, it might cause a pandemic and threaten public health security. In addition to molecular genetic changes in the HA receptor-binding region, NA stalk truncation has also been considered as one of the molecular markers for AIV adaptation to host changes (Matsuoka et al., 2009; Park et al., 2017). Since 2000, the proportion of H5N1 viruses with amino acid deletions at positions 49–68 increased year by year, and even reached 100% in 2007 (Zhou H. et al., 2009). At the same time, we counted the frequency of deletions of NA (49–68) from different host origins during 1997 to 2019. The frequency was the highest in chickens, reaching 98.36% (2231/2268), followed by ducks with 96.19% (1440/1497). The mutation rate of 20 amino acid deletions in wild birds was 88.16% (67/76) (Supplementary Figure 2). The frequency of deglycosylation of AIV isolated from all hosts is very low, showed a 0.2% (12/4857). Therefore, we concluded that NA stalk-deletion of H5N1 is related to poultry adaptability.

The initial understanding of NA truncation was that it could alter its neuraminidase function, and because of the limitation of NA function, could not enable the effective release of offspring viruses, thereby limiting the spread of viruses (Blumenkrantz et al., 2013; Park et al., 2017). In theory, the truncation or deglycosylation of NA stalk is detrimental to virus transmission, while H5N1 may have adapted to changes in NA stalk truncation or deglycosylation by balancing the functions of HA and NA (Zhou H. et al., 2009). This may be compensated by NA itself or other viral proteins for changes in inadequate viral release capacity for progeny virus. This deletion may have negative effects on neuraminidase function, while the virulence enhancement of the virus is evident (Matsuoka et al., 2009; Park et al., 2017).

The replication kinetics results indicated that the nine strains had good growth and replication capacities on the CEF and DEF cells (Figure 2) and in chicken embryos (data not shown), however, rSNA+ and rSNA-Δ50 showed better replication capacities in chicken embryos than the other recombinant viruses (data not shown), indicating that the ability of the viruses to replicate *in vitro* is affected by the control of the glycosylation sites, in addition to the length of the NA. Partial alterations in the NA stalk did not affect viral growth in the tissues and cells, however, the complete absence of the stalk presented a clear attenuated phenotype (Castrucci and Kawaoka, 1993; Li et al., 2017). The balance of HA and NA is an important factor for maintaining virus proliferation, and alterations in NA length will rebalance this activity. The simultaneous presence or absence of four glycosylation sites (at residues 50, 58, 63, and 68) did not affect the infection of recombinant viruses in MDCK cells, while truncation of the NA stalk reduced the infection ability of the viruses (Figure 3), indicating that the HA/NA function balance of viruses with long-stalked NA is better than that of short-stalked viruses (Wagner et al., 2002; Murakami et al., 2008). Although short-stalk and deglycosylated NA viruses had smaller plaque diameters on MDCK cells and showed a decreased neuraminidase activity, the results of increased virulence *in vivo* are consistent with previous reports (Matsuoka et al., 2009; Park et al., 2017). The pathogenicity of AIV is the result of a multiparametric functional balance including HA receptor affinity, NA neuraminidase activity, and the host immune system (Qin et al., 2018), suggesting that the balance achieved *in vitro* does not allow direct analogy *in vivo*. We speculate that the mechanism responsible for this difference may be due to host factors, which needs further exploration. Our results showed that truncation of the NA stalk can effectively enhance the virulence of H5N1 subtype virus to ducks, while the virulence was not positively correlated with the length of truncation, for instance, rSNA-5, rSNA-15, and rSNA-20 were more virulent to ducks than rSNA-10. Deglycosylation was less pronounced than truncation in the promotion of virulence, however, it also can increase the virus shedding rate (Table 5). Although the virus shedding results showed that rSNA-Δ50-Δ58-Δ63-Δ68 with four glycosylation deletions exhibited the highest shedding rate in deglycosylation viruses, it could not indicate that the increase of shedding rate was associated with the number of glycosylation site deletions, as the shedding rates of the other three deglycosylation modes viruses (rSNA-Δ50, rSNA-Δ50–Δ58, and rSNA-Δ50–Δ58–Δ63) were not significantly different. Collectedly, although both deglycosylation and truncation of NA stalk can enhance the virulence of H5N1 subtype virus, truncation is significantly more virulent than deglycosylation.

The stability results explained that the short-stalked NA viruses were more resistant to environmental changes than the long-stalked NA viruses, which might also be one of the reasons why short-stalked NA viruses had become the dominant prevalent strains currently. Neuraminidase activity affects the rate of viral dissociation from erythrocytes. rSNA+, rSNA-5, and rSNA-Δ50 had a higher neuraminidase activity, thus their dissociation rates were also faster. Previous study found that the existence of the glycosylation site at HA 158 enables the virus to release easily from its receptor and enabled reduced dependence on neuraminidase activity (Baigent and McCauley, 2001). Therefore, the release of virus is also influenced by the equilibrium of HA and NA. Parental virus SY strain was used to construct recombinant viruses that had a 158 glycosylation site, which resulted in all viruses can be released within 10 h despite there was a significant difference in neuraminidase activity (Figure 5).

The stalk-truncated H5N1 virus was highly pathogenic to mallard ducks and causes neurological symptoms, which is consistent with previous reports (Lndt et al., 2009; Tang Y. H. et al., 2009). In this study, the oropharyngeal swab titer of the recombinant viruses after the intranasal infection was significantly higher than that from the cloaca swabs, as previously observed (Pantin-Jackwood et al., 2013), which is also consistent with the pattern of viral shedding through the respiratory tract in infected ducks (Germeraad et al., 2019). There are two main modes of horizontal transmission of AIV, including aerosol-mediated airborne transmission and ingestion-based fecal-oral transmission. Therefore, we measured the virus shedding rates in the oropharyngeal and cloaca, the results showed that the NA stalk-deletion and deglycosylation virus were significantly higher than the intact stalk virus. This suggested that the AIV with NA stalk truncation or deglycosylation may be more advantageous for viral transmission in ducks. All the recombinant viruses had high replication titers in the lungs and caused severe pathological damage in the infected ducks. At the same time, the viruses also caused different degrees of damage to other tissues. The results of the brain histopathology also confirmed the appearance of neurological symptoms, showing that the H5N1 AIV infection was mainly caused by respiratory tract injury but was accompanied by systemic symptoms (Stech et al., 2015). In general, both the *in vivo* replication titers and the histopathological results were apparent in demonstrating that the truncation of the NA stalk was more effective than deglycosylation in enhancing the virulence of H5N1 AIV to mallard ducks, which caused more severe pathological damage to the host.

The innate immune response is an important role in the infection process of HPAIV H5N1. The virus will be recognized by the innate immune system, followed by activating antiviral signaling pathways and producing cytokines (Saito et al., 2018). Cytokines are important for regulating local or systemic immune and inflammatory responses (Chen et al., 2018). Cytokines can not only exert immunomodulatory and antiviral effects, but also participate in the occurrence of many diseases under certain conditions. The cytokine storm characterized by excessive inflammatory response caused by HPAIV can lead to an increase in the morbidity and mortality of hosts. H5N1 AIV is a strong inducer of various cytokines and chemokines (IFN-α, IL-6, IL-8, and IL-1β) (Peiris et al., 2009; Saito et al., 2018). It has been found that the mutations in NS1, HA, PB2, and NA genes of AIVs can stimulate a cytokine storm by accelerating virus replication, increasing invasiveness, and promoting the host’s resistance to antiviral responses. Therefore, we measured these cytokines to verify whether the enhanced virulence phenotype induced by NA stalk deletion was caused by a stronger cytokine storm. CD8^+^ T lymphocytes and macrophages are important immune cells. They mainly produce pro-inflammatory cytokines IL-1β, IL-8, and IL-6. In our study, there was no significant difference between IL-1β and IL-6 after challenge with different recombinant viruses. In terms of IL-8, the four strains with the short-stalk group at 3 d.p.i. increased significantly compared with deglycosylation AIVs and rSNA+ (Figures 6B,D,E). At the same time, there was no statistical difference in the transcription level of the IL-10, an important anti-inflammatory cytokine (Figure 6F). The results of the determination of antiviral genes IFN-α and Mx1 showed that short-stalk NA virus can significantly increase the transcription of antiviral genes *in vivo* (Figures 6A,C). The results of cytokine determination indicated that the truncation of NA stalk cannot only increase the pro-inflammatory cytokines, but also induce more antiviral-related genes, implying the complexity of the host–virus relationship after AIV infection. For instance, in the mouse model, the plasmacytoid dendritic cells (pDCs) has been shown to be the main source of type I interferon during AIV infection (Jewell et al., 2007), the effective detection of AIV by pDCs can improve the quality of the immune response and induce a more effective antiviral level at early stage, however, it also can limit the T cell response by inhibiting the Fas-dependent apoptotic pathway, leading to an increase in mortality from lethal infections (Langlois and Legge, 2010). In general, NA deletion can increase the expression of inflammation-related cytokines and antiviral related cytokines, while, IL-10, a cytokine related to inflammation suppression, has no significant increase. This may imply that the truncation of the NA stalk caused the host to produce a strong antiviral response, resulting in uncontrollable the expression of inflammation-related factors, which further exacerbated the irreversible damage to the host before the virus is cleared. Eventually, a phenotype with enhanced pathogenicity was exposed.

In summary, the deletion of the NA stalk region can enhance the virulence and replication titer of AIV, enhance the innate immune response, and trigger host clinical symptoms. The effects of the NA stalk deletion described above are not entirely dependent on the absence of the glycosylation sites in the stalk region. Although the relationship between deglycosylation and virulence is less than NA stalk deletion, the lack of glycosylation sites has a significant impact on virus shedding.

## Data Availability Statement

The raw data supporting the conclusions of this article will be made available by the authors, without undue reservation.

## Ethics Statement

The animal study was reviewed and approved by the Jiangsu Province Administrative Committee for Laboratory Animals (approval number: SYXK-SU-2017-0007) and complied with the guidelines of Jiangsu Province Laboratory Animal Welfare and the Ethics of Jiangsu Province Administrative Committee of Laboratory Animals. Written informed consent was obtained from the owners for the participation of their animals in this study.

## Author Contributions

SC, KQ, DW, and DP conceived and designed the experiments. SC and DW performed the experiments. SC, KQ, and TQ analyzed the data. SC, KQ, DW, and YD contributed reagents, materials, and analysis tools. SC, KQ, DP, and XL wrote the manuscript. All authors contributed to the article and approved the submitted version.

## Conflict of Interest

The authors declare that the research was conducted in the absence of any commercial or financial relationships that could be construed as a potential conflict of interest.

## Footnotes

1 www.gimp.org
