# Supplementary material for: Truncation or Deglycosylation of the Neuraminidase Stalk Enhances the Pathogenicity of the H5N1 Subtype Avian Influenza Virus in Mallard Ducks
Source: Front Microbiol. 2020 Oct 22;11:583588. doi: 10.3389/fmicb.2020.583588 (PMC7641914; doi:10.3389/fmicb.2020.583588)
Supplement: Supplementary file 1 [file Data_Sheet_1.docx]

Supplementary Material

# Supplementary Figures and Tables

## Supplementary Figures
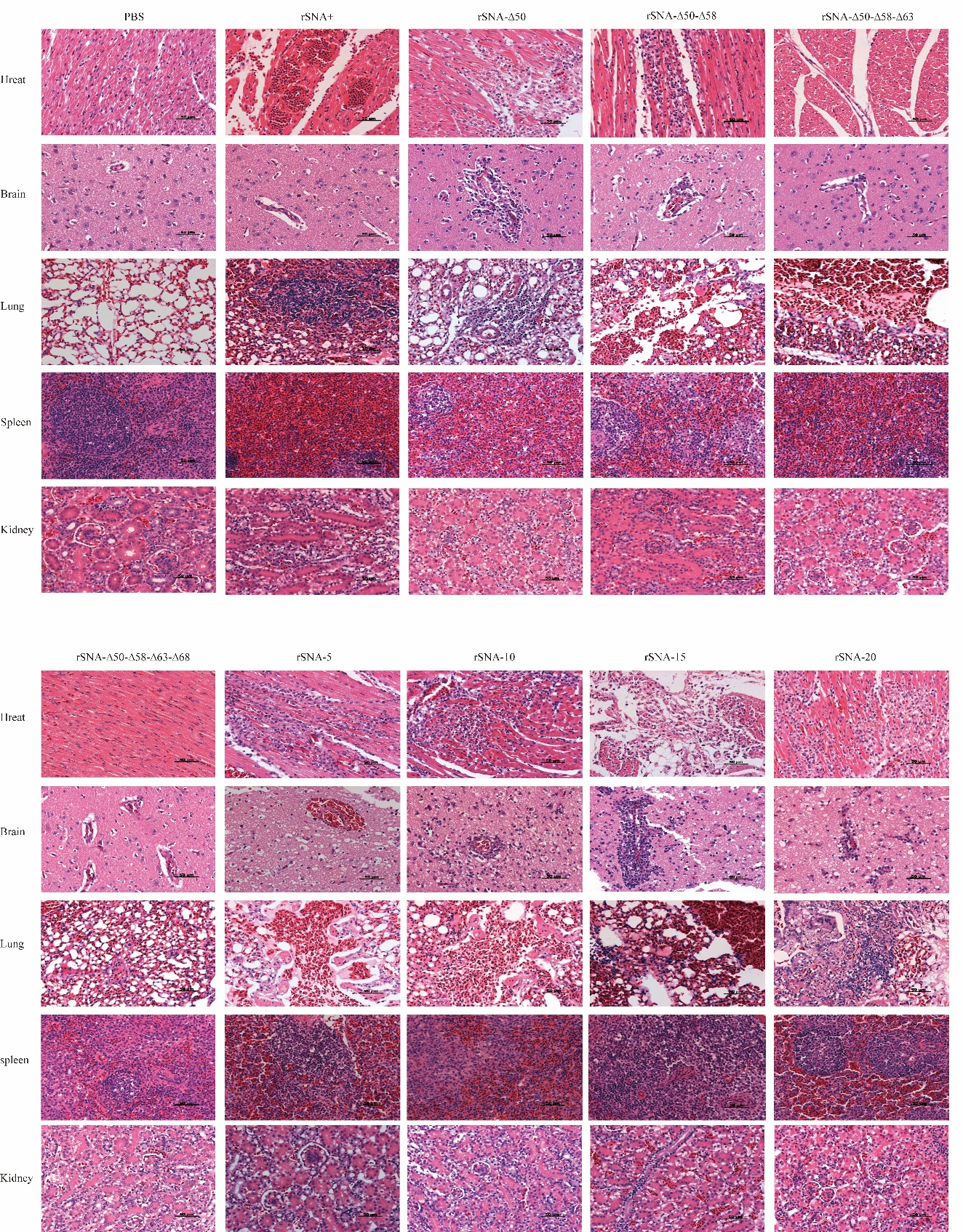


**Supplementary Figure 1.** Histopathologic changes in the heart, brain, lung, spleen and kidney of virus-infected ducks (50 μm).

Ducks (n=3) were intranasally infected with 106.0 EID50 of recombinant viruses. At 5 d. p. i., three ducks from each group were euthanized. Hearts, brains, lungs, and spleens were collected for paraffin section and hematoxylin and eosin stain. Scale bar, 50 μm.

**Supplementary Figure 2.** The occurrence rate of NA stalk deletion in different hosts. Statistics of 4,857 H5N1 AIVs from avian in the CISAID database from 1997 to 2019, including 2268 chickens, 1497 ducks, 200 geese, and 76 wild birds.
